# Primary follicular lymphoma of the duodenum: a case report and review of literatures 

**Published:** 2021

**Authors:** Cheep Charoenlap, Keerati Akarapatima, Komsai Suwanno, Attapon Rattanasupar, Arunchai Chang

**Affiliations:** 1 *Department of Anatomical Pathology, Hatyai Hospital, Songkhla, Thailand*; 2 *Division of Gastroenterology, Department of Internal Medicine, Hatyai Hospital, Songkhla, Thailand*; 3 *Division of Hematology, Department of Internal Medicine, Hatyai Hospital, Songkhla, Thailand*

**Keywords:** Follicular lymphoma, Duodenum, Lymph node, Non-Hodgkin lymphoma

## Abstract

Follicular lymphoma (FL) is one of the most common types of non-Hodgkin lymphoma (NHL). The gastrointestinal tract is the most involved extra-nodal site of NHL. Primary duodenal FL (DFL) is a rare entity with only a few reported cases. It mainly involves the second part of the duodenum and has an excellent prognosis. We report the case of a 74-year-old man who underwent esophagogastroduodenoscopy. Endoscopic findings revealed multiple small whitish mucosal nodules which were detected around the major duodenal papilla. Biopsy of these lesions was compatible with grade I FL. Further investigation failed to demonstrate any evidence of nodal or systemic involvement; thus, the clinical staging was stage I, according to the Lugano staging system. A “watch and wait” policy was chosen. Neither lesion aggregation nor lymphadenopathy was noted during the 5-year follow-up period. In conclusion, this was an uncommon case of DFL with an indolent nature and excellent prognosis. However, further studies are needed to clarify the characteristics, prognosis, and therapeutic approach.

## Introduction

 Follicular lymphoma (FL) is a neoplasm of germinal center B cells and usually involves the lymph nodes. FL is one of the most common types of non-Hodgkin lymphoma (NHL) with high incidence rates in Europe and the United States. Moreover, it is the second most common type of NHL in Japan (1, 2). The gastrointestinal (GI) tract is the most commonly involved extra-nodal site of NHL; however, primary duodenal FL (DFL) is a rare entity with only a few reported cases. It was a newly recognized entity in the 2016 World Health Organization (WHO) classification update (3). The second part of the duodenum is the most frequently involved site and is reported to have an excellent prognosis, even without any treatment, which is different from the prognosis of nodal FL with GI involvement.(2) Herein, we report the case of a 74-year-old man with primary DFL who had typical endoscopic and histological results. 

## Case Reports

**Figure 1:**
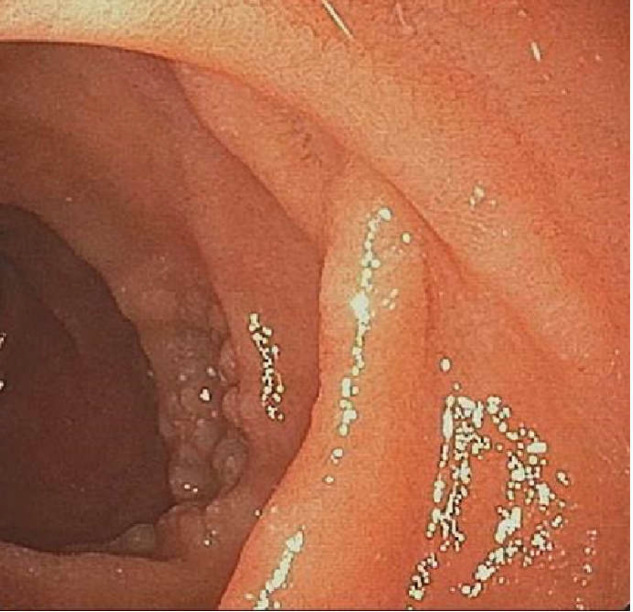
Endoscopy shows whitish multi-nodular mucosal lesions around the major papilla, with a diameter of 0.1-0.5 cm

A 74-year-old male patient underwent esophagogastroduodenoscopy (EGD) for the evaluation of epigastric discomfort. He had no relevant medical history. Physical examination was unremarkable, and there was no evidence of hepatosplenomegaly or superficial lymphadenopathy. All laboratory findings, including lactate dehydrogenase, were within normal ranges. Endoscopically, multiple small whitish mucosal nodules were detected around the major duodenal papilla (Figure 1). 

**Figure 2:**
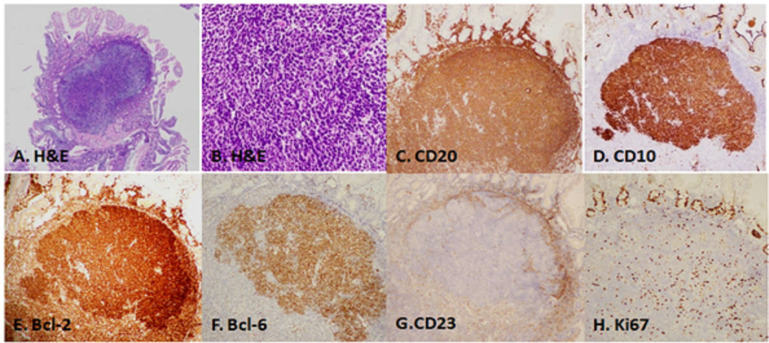
A polypoid lesion showing a prominent germinal center in the mucosa. (A) Atypical lymphoid aggregation composed of a germinal center of mainly uniform, small cleaved B-lymphoid cells without tingible body macrophages. (B) The tumor cells were immunopositive for CD20 (C), CD10 (D), Bcl-2 (E), and Bcl-6 (F). (G) CD23 positive follicular dendritic cells were seen at the periphery of the neoplastic germinal center. (H) The Ki67 reactivity rate was low

Biopsy of these lesions revealed atypical lymphoid aggregation composed of a germinal center of mainly uniform, small cleaved B-lymphoid cells without tingible body macrophages. The tumor cells were immunopositive for CD20, CD10, Bcl-2, and Bcl-6. Ki67 reactivity rate was low. CD23 positive follicular dendritic cells were seen at the periphery of the neoplastic germinal center (Figure 2). Furthermore, no evidence of *Helicobacter pylori* was detected by the rapid urease test or histological findings of random gastric biopsy. Colonoscopy revealed no abnormality. A complete enteroscopy, using double-balloon enteroscopy or video capsule endoscopy, was proposed, but it was not performed as the patient refused to undergo the procedure. Computed tomography (CT) of the neck, chest, abdomen, and pelvis along with bone marrow examination were performed but failed to demonstrate any evidence of nodal or systemic involvement. Finally, a diagnosis of primary FL of the duodenum was made. The clinical staging was considered to be stage I, according to the Lugano staging system. After discussion with the patient, a “watch and wait” policy was chosen, and no local or systemic treatment was given. EGD and CT were performed annually. Neither lesion aggregation nor lymphadenopathy was noted during the 5-year follow-up period.

## Discussion

FL is one of the most common types of NHL (3). However, primary GI FL has been recognized as a relatively rare entity, accounting for 1%-3.6% of NHL cases with primary GI involvement (4).

The most frequently involved sites are the second part of the duodenum (81%), particularly the peri-ampullary area, followed by the jejunum (40%) (5, 6). Eighty-one percent of patients with DFL had a synchronous jejunal or ileal lesion at the time of diagnosis. Thus, endoscopic evaluation throughout the GI tract is recommended (6). The typical endoscopic finding is a whitish multinodular mucosal or polypoid lesion with a diameter usually between 0.1 and 0.5 cm. These lesions can become ulcerated and have the potential to develop multifocal involvement of the GI tract (7). Takeuchi et al. speculated that these whitish lesions reflect the infiltration of the lymphoma cells within the villi, and biopsy samples should be obtained from not only the erosions or ulceration, but also from the surrounding whitish mucosa with enlarged villi (8).

**Table 1:** Patients with stage I-II DFL and disseminated nodal disease

Patient number	Reference	Age (year)	Sex	Stage	Initial management	Duration to PD	Site of involvement^a^	Second management^b^	Response	Time from PD^c^
1	Sentani et al.^1^	50	Female	I	Irradiation	4 months	Submandibular gland and lymph node	N/A	CR	58 months
2	Nehme et al.^9^	51	Female	I	Rituximab	62 months	Multiple mesenteric nodes and bilateral lymph node enlargement	Rituximab	CR	3 years
3	Schmatz et al.^10^	N/A	N/A	I	Watch and wait	5 years	Mesenteric nodes	Rituximab+CHOP+radiation	CR	35 months
4	Schmatz et al.^10^	N/A	N/A	I	Watch and wait	5 years	Mesenteric, axillary and cervical nodes	Rituximab+bendamustine	CR	41 months
5	Iwamuro et al.^11^	52	Female	I	Pancreatoduodenectomy	11 years	Jejunum with multiple intra-abdominal lymph node enlargement	Watch and wait	N/A	N/A
6	Mori et al.^14^	N/A	N/A	II_2_	Watch and wait	7.5 months	Para-aortic nodes	Rituximab+CHOP	CR	54.7 months
7	Seki et al. ^18^	41	Female	I	Watch and wait	45 months	Inguinal node	Rituximab	CR then PD*	81 months
8	Akamatsu et al.^19^	54	Female	II_2_	Watch and wait	6 months	Abdominal lymph node enlargement	Rituximab+CHOP	CR	2.5 years

CR, complete remission; PD, progressive disease; CHOP, a combination chemotherapy with cyclophosphamide, doxorubicin, vincristine, and prednisone

*Recurred in the stomach, rectum, and mesenteric and external iliac nodes at 81 months after the second management; then rituximab was administered. The patient was in complete remission at 15 years after the initial presentation. ^a ^Areas in which disease progression was detected by endoscopy, radiology, or tissue biopsy after initial management ^b ^Treatments or procedures performed on patients after disease progression^c ^Duration between complete second management to achieved CR at last follow-up or detectable disease progression

**Table 2:** Patients with stage 1 DFL and high grade B-cell transformation

Patient number	Reference	Age (year)	Sex	Stage	Initial management	Duration to PD	Site of involvement^a^	Second management^b^	Response	Time from PD^c^
1	Sentani et al.^1^	N/A	N/A	I	N/A	4 months	N/A	N/A	N/A	N/A
2	Mori et al.^14^	N/A	N/A	I	Watch and wait	4.7 months	Cervical node	CHOP+radiation	CR	6 years
3	Miyata-Tanaka et al. ^20^	73	Male	I	Watch and wait	62 months	Bone marrow and intestine	Chemotherapy and partial intestinal resection	N/A	N/A
4	Akiyama et al.^21^	46	Female	I	Watch and wait	7 years	Bone marrow with multiple lymphadenopathy	Rituximab+CHOP	CR	2.5 years
5	Kitabatake et al.^22^	71	Male	I	Watch and wait	5.5-6 years	Mesenteric lymph nodes	Rituximab+CHOP	CR	7 years
6	Hangai et al.23	45	Male	I	Rituximab+CHOP	At diagnosis	Duodenum	-	CR	N/A
7	Shia et al.^24^	44	Female	I	Chemotherapy	At diagnosis	Duodenum	-	CR	10 months
8	Tanigawa et al.^25^	52	Male	I	Watch and wait	7.6 years	Multiple abdominal lymph nodes	Rituximab+CHOP Rituximab+ESHAP+radiation	Progression/Death	7 months

CR, complete remission; PD, progressive disease; CHOP, a combination chemotherapy with cyclophosphamide, doxorubicin, vincristine and prednisone; ESHAP, etoposide, cytarabine, cisplatinum, and methylprednisolone.

a Areas in which disease progression was detected by endoscopy, radiology, or tissue biopsy after initial management. ^b ^Treatments or procedures performed on patients after detection of disease progression. ^c ^Duration from last follow-up or detection of disease progression to completion of second management where CR is achieved.

Pathologically, DFL shows features similar to a nodal lymphoma, which is characterized by several well-circumscribed germinal centers packed together with centrocytes and rare centroblasts with no visible tingible body macrophages and without mantle zones. The sheets of small lymphoid cells with an irregular nuclear contour are usually present outside the follicular structures, and the lymphoma usually involves the mucosa and submucosa. The main differential diagnosis is a reactive germinal center. Unlike reactive germinal centers where the lymphoid follicles are composed of a heterogeneous population of centrocytes, centroblasts, tingible body macrophages and dendritic cells, the normal immunoarchitecture of a germinal center is highlighted by the lack of expression of BCL2 within the germinal center B cells (3). The neoplastic cells show an immunophenotype similar to that of a low-grade nodal FL by expressing CD20, CD79a, CD10, BCL-6, and BCL-2, and they lack the expression of CD3, CD5, CD23, cyclin D1, and MUM-1. They also have a low Ki-67 proliferation rate.(3)

DFL has an immunophenotype similar to that of other FLs and usually carries the typical t(14;18)(q32;q21)(IGH/*BCL2*) translocation, but the gene expression pattern of DFL shares more similarities with that of *H. pylori*-associated gastric MALT lymphoma than with that of nodal FL. According to the validation of microarray data by quantitative reverse transcription-polymerase chain reaction for CCL20 and MADCAM1, DFL and MALT lymphoma have increased expressions of CCL20 and MADCAM1, contrary to the low levels expressed in nodal FL. In a study using array comparative genomic hybridization, although a duodenal-type FL is positive for the IGH/*BCL2 *translocation, it has a lower frequency of other genetic aberrations than nodal FL (3). Takata et al. suggested that CCL20 and CCR6 co-expression may also be involved in DFL tumorigenesis, and the inflammatory background and antigen stimulation may underlie the pathogenesis of DFL. Furthermore, the immunohistochemical results for the PCDHG family, which consists of PCDHGA3, PCDHGA8, and PCDHGB4, revealed that they are frequently expressed in DFL and NFL, whereas in MALT lymphoma, they are less frequently expressed. However, no reports have demonstrated that PCDHG family proteins are overexpressed in malignant lymphoma (6).

In contrast to other extranodal involvements which usually occur in disseminated nodal diseases, DFL seems to share some common characteristics with nodal diseases, such as an indolent nature and excellent prognosis.(10) DFL rarely causes clinical symptoms, and patients with DFL are normally diagnosed with a low-grade (grades 1-2) lesion at the early stage of the disease (2, 7, 9). However, Schmatz et al. described a series of 63 cases of DFL with an overall median follow-up duration of 77 months; only 2 out of 24 untreated patients developed a nodal disease. The remaining 61 patients had no extranodal disease or large cell transformation (10). Sentani et al. revealed that only 1 out of 14 DFL cases experienced a relapse in the submandibular gland and lymph nodes at 4 months after the initial treatment (1). Only a few DFL cases with a disseminated nodal relapse have been reported (Table 1). The occurrence of high-grade transformation is infrequent but possible, (9) and only seven cases of DFL with diffused large B cell lymphoma transformation have been reported (Table 2).

Several treatment options for GI-FL have been proposed, such as surgical resection, chemotherapy, and radiation therapy; however, no consensus regarding the choice of treatment for DFL has been established yet. The clinical course of DFL tends to be indolent, and it has an excellent long-term survival rate, even with local recurrence in the intestine, and low risk of progression to a nodal disease (11). A recent multicenter study demonstrated that the median survival of intestinal FL, mostly DFL, is 10 years after diagnosis (12). Data from this study showed that male sex and the presence of abdominal symptoms were independently associated with poor progression-free survival. In contrast, the presence of duodenal involvement was an independent predictive factor for a favorable clinical course. Several authors mentioned that the “watch and wait” approach would be a suitable option, based on the results of their case series, (10, 13-15) and this approach was generally applied to DFL patients without systemic or nodal involvement (16). The 2016 WHO classification update summarized that patients with DFL have excellent long-term survival, even with local recurrence in the intestine, owing to the low risk of progression to a nodal disease (3). Furthermore, a recent prospective study conducted by Tari et al. showed that the “watch and wait” strategy is a treatment option comparable to chemotherapy for asymptomatic intestinal FL (17). However, a large-scale study with a longer follow-up duration is required to evaluate which treatment option is suitable for patients with this disease entity.

In conclusion, we present the case of a 74-year-old male patient with primary DFL who had classical endoscopic and histologic results. The patient also underwent staging investigations, including laboratory diagnosis, whole-body CT, and bone marrow biopsy to exclude an involvement of nodal FL, which had already occurred in the advanced stage. No evidence of nodal or systemic involvement was identified. The patient was assigned to a “watch and wait” policy and showed excellent outcomes during the follow-up. However, further studies are needed to clarify the characteristics, prognosis, and therapeutic approach of this rare entity.
